# To W. O., on His Inquiries in the Last Number

**Published:** 1815-01

**Authors:** C. Y. Collec


*For the Medical and Physical Journal.*

### *To* W. O., *on his Inquiries in the last Number.*

**Morbi haud ignarus miseris succurrere disco.**

SIR, AND FELLOW-SUFFERER,

I HAVE read with real sympathy your affecting history, and should not do justice to my own feelings if I delayed giving you at least one comfort, in assuring you, that I have gone through most of what you complain and am now in good health, though not without occasional pains at the change of weather. Having

Having, like yourself, enjoyed the advantage of a liberal education, I read as you have done, and now I will tell you my authors, and the manner in which I availed myself of them. Respecting the first, most probably your account would be similar to mine; but, by the tenor of your letter, the latter does not appear to have occurred to you.

It cannot be necessary to mention the late Mr. Hunter. I read his treatise at first, before Dr. Adams had published the edition with his own commentaries. This, when it appeared, led me by reference to his Morbid Poisons, and the references there led me to all the other writers on that strange disease, and the various others with which it is confounded or complicated. There is nothing in either of these works that a well-educated man with leisure and patience may not understand. Mr. Hunter, though extremely dull in his style, has introduced a manner of writing intelligible to every reader; and such of his followers as are not well understood, are only obscure from the difficulty of making up their minds on subjects in which they are unwilling to confess their ignorance. This you will easily perceive if you read with as much judgment as you write.

As I read each author, I selected those passages which applied most to my own case; and if not satisfied with their account, wrote to them for further information, inclosing my fee, a measure which the present currency very much facilitates. I consulted none verbally, but chose to have their opinions in writing, by which they were forced to think, and I had leisure to compare their accounts. Not satisfied with mere advice and prescriptions, I always required the application of that advice to their various writings, and by these means soon discovered whether they were as firm in their decisions as they pretended to be, and whether that firmness arose from experience and conviction, or was only an outside show of superior knowledge. In the last case my learned correspondent soon dropt writing, or answered me in so petulant a manner as induced me to drop all further communication with him. Be particularly aware of those who use hard words. I was told of *necrosis*, and, presuming it could only be a Greek term for mortification, expressed my uneasiness. The answer was evasive, and I never could gain a satisfactory explanation of the word. Have you been more fortunate?

To be short, sir, I found ample satisfaction in one rational being who seemed pleased at my multiplied inquiries, and flattered at the manner in which I questioned him concerning his own writings. By pursuing a similar course, I have no doubt of your succeeding, like myself; and, with every wish for such success, remain, &c.

